# Structural and Genetic Studies Demonstrate Neurologic Dysfunction in Triosephosphate Isomerase Deficiency Is Associated with Impaired Synaptic Vesicle Dynamics

**DOI:** 10.1371/journal.pgen.1005941

**Published:** 2016-03-31

**Authors:** Bartholomew P. Roland, Alison M. Zeccola, Samantha B. Larsen, Christopher G. Amrich, Aaron D. Talsma, Kimberly A. Stuchul, Annie Heroux, Edwin S. Levitan, Andrew P. VanDemark, Michael J. Palladino

**Affiliations:** 1 Department of Pharmacology & Chemical Biology, University of Pittsburgh School of Medicine, Pittsburgh, Pennsylvania, United States of America; 2 The Pittsburgh Institute for Neurodegenerative Diseases (PIND), University of Pittsburgh School of Medicine, Pittsburgh, Pennsylvania, United States of America; 3 Department of Biological Sciences, University of Pittsburgh, Pittsburgh, Pennsylvania, United States of America; 4 Energy Sciences Directorate/Photon Science Division, Brookhaven National Laboratory, Upton, New York, United States of America; University of Washington, UNITED STATES

## Abstract

Triosephosphate isomerase (TPI) deficiency is a poorly understood disease characterized by hemolytic anemia, cardiomyopathy, neurologic dysfunction, and early death. TPI deficiency is one of a group of diseases known as glycolytic enzymopathies, but is unique for its severe patient neuropathology and early mortality. The disease is caused by missense mutations and dysfunction in the glycolytic enzyme, TPI. Previous studies have detailed structural and catalytic changes elicited by disease-associated TPI substitutions, and samples of patient erythrocytes have yielded insight into patient hemolytic anemia; however, the neuropathophysiology of this disease remains a mystery. This study combines structural, biochemical, and genetic approaches to demonstrate that perturbations of the TPI dimer interface are sufficient to elicit TPI deficiency neuropathogenesis. The present study demonstrates that neurologic dysfunction resulting from TPI deficiency is characterized by synaptic vesicle dysfunction, and can be attenuated with catalytically inactive TPI. Collectively, our findings are the first to identify, to our knowledge, a functional synaptic defect in TPI deficiency derived from molecular changes in the TPI dimer interface.

## Author Summary

Glycolysis is the metabolic pathway that cells use to break down the sugar glucose, and mutations in the genes that control the glycolytic pathway elicit a collection of diseases known as glycolytic enzymopathies. Glycolytic enzymopathies are rare genetic diseases that lead to the degeneration of patient red blood cells. *Triosephosphate isomerase* is a gene that encodes a part of this glycolytic process, and patients with mutations in this gene experience the typical blood disorder, as well as severe neurologic dysfunction and often infant death. Until now, a molecular source of neurologic dysfunction in *triosephosphate isomerase* mutants was unknown. We have discovered that mutations that disrupt the self-association of the triosephosphate isomerase enzyme lead to neurologic dysfunction in fruit flies. This neurologic dysfunction is characterized by the abnormal cycling of neuronal vesicles that contain neurotransmitters. Given the evolutionary conservation of *triosephosphate isomerase* and neuronal synaptic function, we believe that these observations represent the dysfunction seen in human patients.

## Introduction

Triosephosphate isomerase (TPI) is a glycolytic enzyme that converts dihydroxyacetone phosphate (DHAP) into glyceraldehyde-3 phosphate (GAP). TPI is a non-linear member of the glycolytic pathway, enhancing the efficiency of the catabolic process, and several missense mutations within *TPI* lead to a disease known as TPI deficiency [1]. TPI deficiency is one member of a group of disorders caused by mutations in glycolytic enzymes, collectively called glycolytic enzymopathies. Glycolytic enzymopathies are largely characterized as blood disorders, with all patients experiencing hemolytic anemia [2,3]. TPI deficiency is one of the few glycolytic diseases associated with patient neurologic dysfunction, and by far the most severe [1,4]. Clinical examinations of TPI deficiency patients have established that this disease is often characterized by episodic seizures, periodic dystonia, and progressive weakness and flaccidity in extremities [5–11]. Cellular studies have centered on erythrocytes and lymphocytes, leaving it unclear how TPI molecular dysfunction influences the nervous system. Further, the absence of neurologic dysfunction in many other glycolytic enzymopathies has made it unclear whether these symptoms are related to glycolysis or an as-yet-unidentified function of TPI.

Several reports have suggested that TPI deficiency is a disease caused by changes in protein conformation rather than metabolic defects [4,12–14]. A human protein structure and yeast genetic studies have asserted that defects in TPI dimerization are the primary determinants of pathology [15,16], revealing no catalytic defects *in vitro* or from cell lysate. However, not all TPI deficiency mutations lack catalytic defects. An erythrocyte study examining two Hungarian brothers with identical *TPI* alleles revealed equivalent reductions in TPI activity in both individuals [17], yet one exhibited severe neurologic dysfunction and the other was asymptomatic. Further, a recent structural study demonstrated that the hTPI^I170V^ substitution significantly altered enzyme kinetics and protein stability through a molecular alteration near the catalytic pocket [18]. Collectively, each of these studies failed to establish a causal relationship between TPI activity and disease.

Previously, a pathogenic substitution in *Drosophila TPI* (*dTPI*^*M80T*^) was identified, eliciting mechanical- and thermal-stress dependent paralysis [19,20]. These behavioral phenotypes have been independently established as hallmarks of neurologic dysfunction and each has been used in forward genetic screens to identify novel components of neuronal transmission [21–23]. The *dTPI*^*M80T*^ allele was identified in such a screen [20,24], and to-date *D*. *melanogaster* is the only model organism to exhibit neurologic dysfunction caused by TPI deficiency. The dTPI^M80T^ protein was found to be prematurely degraded with reduced catalytic activity [25,26]. This reduction in catalytic activity was shown to inhibit glycolytic flux as well as induce metabolic stress [19,25], yet did not change ATP levels *in vivo* [20]. Subsequent studies demonstrated that the *dTPI*^*M80T*^ point mutation could be complemented by the addition of a catalytically inactive *TPI* without increasing lysate isomerase activity or alleviating metabolic stress [25], suggesting that dTPI^M80T^ may elicit pathology through a change in protein conformation. Thus, we initially examined the molecular source of *dTPI*^*M80T*^ pathology.

To determine whether *Drosophila* TPI deficiency was caused by changes in protein conformation, we purified and assessed the physical characteristics of hTPI^M82T^, the human equivalent of dTPI^M80T^, and revealed impaired TPI dimerization. These results were further supported when independent alleles bearing mutations at the TPI dimer interface phenocopied the *Drosophila* behavioral dysfunction seen in the *dTPI*^*M80T*^ allele. These experiments provided novel insight into the pathogenesis of TPI deficiency leading to the conclusion that alterations of TPI dimerization are sufficient to elicit neuropathology.

Defining the molecular source of TPI neurologic dysfunction led to the generation of new alleles containing dimer-interface mutations. These novel *TPI* alleles were characterized by extreme behavioral defects, directing new investigations into the neuropathogenic mechanism of TPI deficiency. An examination of vesicle dynamics at the larval neuromuscular junction (NMJ) revealed a severe impairment that appears to be related to vesicle recycling. Further, complementation with a *TPI* allele encoding catalytically inactive TPI rescued both synaptic dysfunction and behavior, thereby characterizing a cellular mechanism of TPI deficiency neuropathology.

Collectively, the results of this study support the conclusion that an improperly formed dimer interface is sufficient to elicit TPI deficiency neuropathology. Further, our experiments establish that a functional synaptic defect occurs in our *Drosophila* model of TPI deficiency.

## Results

### hTPI^M82T^ impairs TPI homodimerization

TPI is a homodimeric enzyme with catalytic sites in the C-terminal of a triose isomerase (TIM) barrel tertiary structural motif [27]. Each catalytic site is rigidified through dimerization to increase catalytic turnover, yet each active site works independently [28–30]. A *dTPI*^*M80T*^ substitution was previously isolated and demonstrated to elicit pathology in a *Drosophila* model of TPI deficiency [19,20]. The *dTPI*^*M80T*^ substitution is physically located in a solvent-exposed region of the protein near the dimer interface [25]. Numerous misfolding events could be hypothesized to occur as a function of the TPI^M80T^ substitution, among them alterations of dimerization [15,16] and aggregation [31]. To examine the structural change elicited by M80T *in vitro* we purified *Drosophila* dTPI^M80T^.

**Fig 1:**
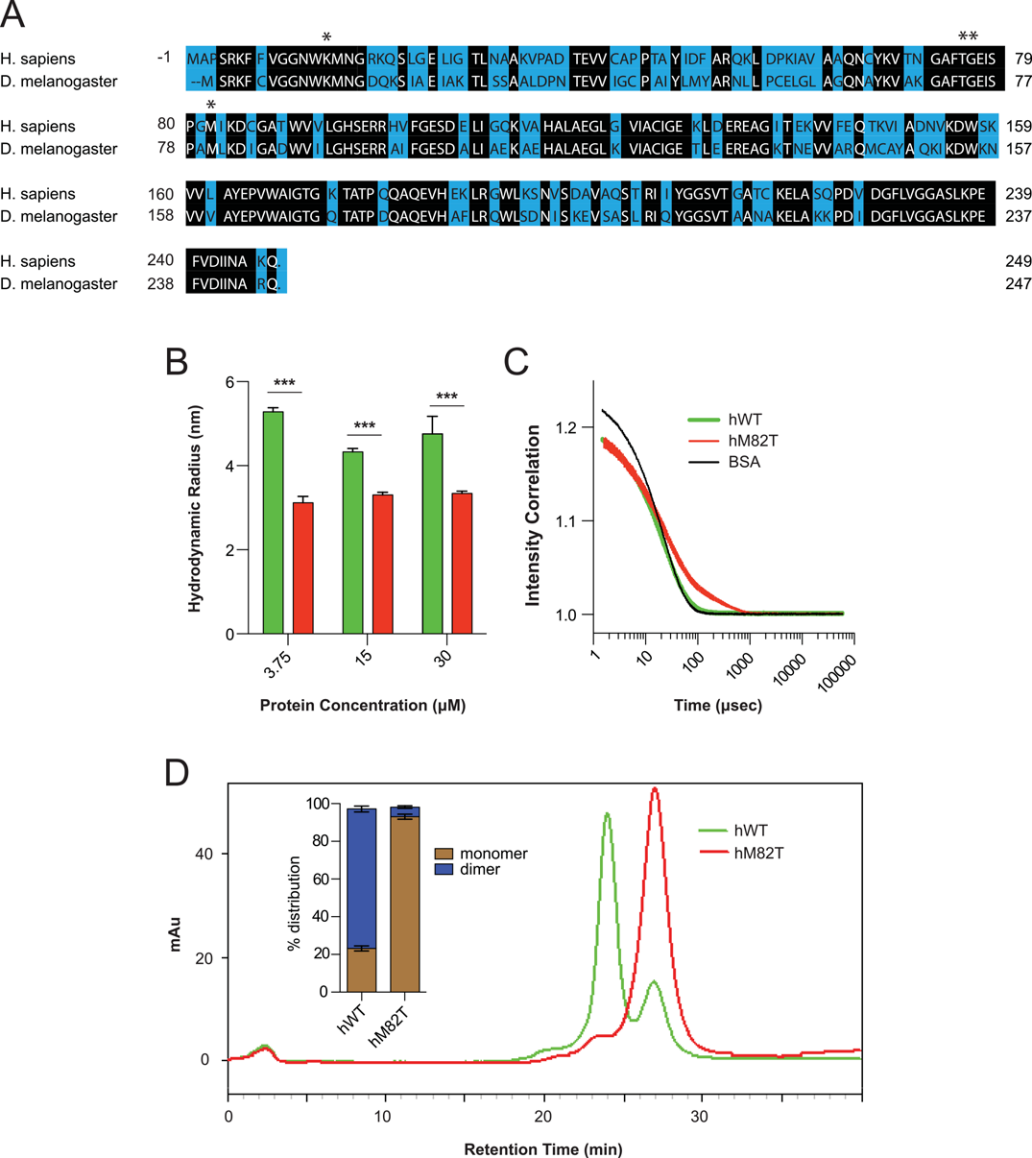
hTPI^M82T^ elicits a conformational change in TPI resulting in reduced dimerization. (A) Sequence alignment of *D*.*melanogaster* and *H*.*sapiens* TPI protein sequence with asterisks highlighting residues of interest. (B) The hTPI^M82T^ mutation confers a reduction in mean protein hydrodynamic radius as measured by dynamic light scattering. (C) Intensity correlation plots reveal a largely monodisperse hTPI^WT^ population and polydisperse hTPI^M82T^ population. (D) Gel filtration indicates a change in monomer:dimer ratios elicited by hTPI^M82T^ with relative quantification (inset). n≥3, comparisons were made using Student’s T test, *** indicates p<0.001.

Previous purification experiments had yielded *Drosophila* TPI enzyme, but these samples proved aggregation prone at high concentrations. Conversely, purified human TPI (hTPI) was well-behaved; therefore, in order to physically characterize TPI we studied the human protein *in vitro*. To validate the use of human protein *in vivo* we generated human WT (*hTPI*^*WT*^) and human M80T (*hTPI*^*M82T*^) alleles in the *Drosophila TPI* gene locus using an established genomic engineering (GE) system [25]; the hTPI^M82T^ substitution is equivalent to dTPI^M80T^ (Fig 1A). We found that *hTPI*^*M82T*^ was able to recapitulate the disease phenotypes observed in *dTPI*^*M80T*^ (S1 Fig). The phenotypes of *hTPI*^*M82T*^ were remarkably similar but less severe than *dTPI*^*M80T*^, possibly due to subtle organism-specific changes in the dimer interface [32]. Confirmation that *hTPI*^*M82T*^ pathologically phenocopied *dTPI*^*M80T*^ indicated that any conformational change elicited by dTPI^M80T^ was likely retained in the human protein.

We utilized dynamic light scattering (DLS) to examine potential conformational differences between hTPI^WT^ and hTPI^M82T^. Analyses of 15 μM solutions of hTPI^WT^ revealed a hydrodynamic radius of 4.3±0.08 nm, while hTPI^M82T^ exhibited a significant reduction to 3.3±0.06 nm (Fig 1B); these results were consistent across two additional protein concentrations, 3.75 μM and 30 μM (Fig 1B). The linear slope generated by plotting the intensity correlation data suggested the hTPI^WT^ sample was largely monodispersed, much like that of the 15 μM sample of bovine serum albumin (Fig 1C). Conversely, hTPI^M82T^ samples exhibited a non-linear slope (Fig 1C), suggesting the possibility of a polydisperse protein population. Polydisperse protein populations indicated the sample was a mixed population in solution, and the observed reduction in TPI mean hydrodynamic radius suggested the sample could be a mixture of monomer and dimer TPI species.

We examined enzyme dimerization by assessing protein size via gel filtration chromatography. A standard curve was used to establish column resolution, 15 μM samples of hTPI^WT^ and hTPI^M82T^ were injected onto the gel filtration column, and their migration monitored by UV light at 280 nm. hTPI^WT^ samples separated into two distinct peaks–one at ~24 min. and another at ~27 min. corresponding to ~50 kDa and ~28 kDa, respectively (Fig 1D). Dimeric and monomeric hTPI are 54 and 27 kDa, respectively. Integrating the peak areas revealed an ~80:20 split in dimer:monomer ratio of hTPI^WT^ (Fig 1D inset). In contrast, the majority of the hTPI^M82T^ sample eluted at 27 min., resulting in a ~5:95 dimer:monomer ratio (Fig 1D inset). These data led us to conclude that the hTPI^M82T^ substitution elicited a dramatic conformational change in TPI resulting in a disruption of dimerization. Interestingly, the gel filtration results did not precisely reflect the monodisperse vs. polydisperse observations of the DLS experiments; we believe this could be due to dilution effects as the proteins migrated over the large gel filtration column.

### TPI dimer interface mutants recapitulate dTPI^M80T^ neuropathology

**Fig 2:**
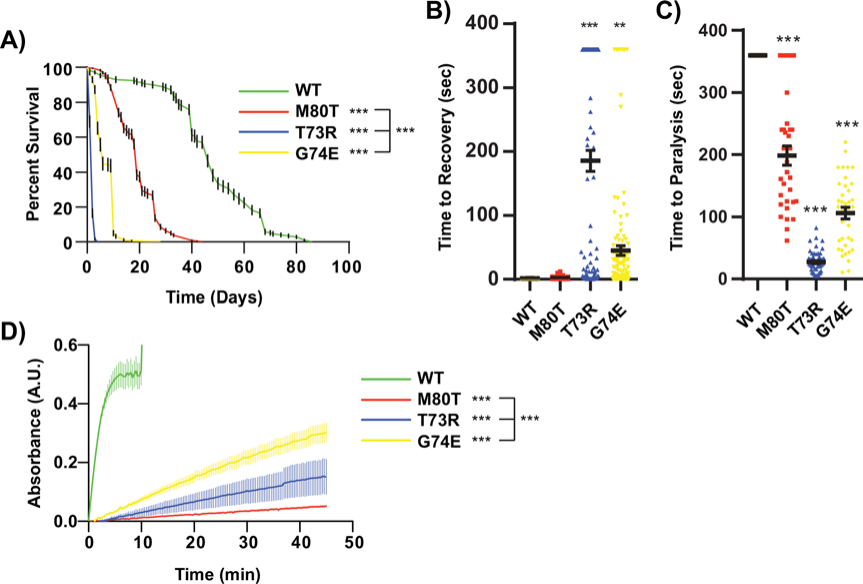
Mutations affecting the TPI dimer interface recapitulate *dTPI*^*M80T*^ phenotypes. (A) *dTPI*^*T73R*^ and *dTPI*^*G74E*^ homozygotes display severely reduced lifespans, n>150. (B) Dimer interface mutations exhibit severe mechanical stress at Day 1 and (C) thermal stress sensitivity at Day 2, n>30. Thermal stress paralysis times at 360 sec. represent wild type behavior, the assay was stopped at 6 min. (D) Both *dTPI*^*T73R*^ and *dTPI*^*G74E*^ homozygotes display reduced lysate isomerase activity, n≥3. Comparisons were made with a One-way ANOVA using Tukey’s post hoc test, and lifespans by a Log-rank (Mantel-Cox) survival test, ** indicated p<0.01, *** p<0.001.

Having established that the hTPI^M82T^ mutation alters enzyme dimerization *in vitro*, we sought to assess whether other substitutions at the TPI dimer interface were sufficient to elicit neuropathology. Two novel *TPI* alleles (*dTPI*^*T73R*^ and *dTPI*^*G74E*^) were generated using GE. Our hTPI dimer analyses (S2 Fig) and data from previous TPI studies [28] indicated these substitutions would result in dimer defective TPI. *dTPI*^*T73R*^ and *dTPI*^*G74E*^ dimer interface mutants elicited a more severe pathology than *dTPI*^*M80T*^, and stocks required maintenance over balancer chromosomes due to their poor viability. Test crosses of balanced stocks yielded significantly fewer homozygous animals than the Mendelian predicted 33%, and homozygous animals were extremely short-lived (Fig 2A), with median lifespans of 2 and 5 days for *dTPI*^*T73R*^ and *dTPI*^*G74E*^, respectively.

Mechanical- and thermal stress-dependent behavioral defects were assessed at Day 1 and Day 2, respectively, as these phenotypes have been demonstrated to be hallmarks of *Drosophila* TPI deficiency [19,20,25,26,33,34]. *dTPI*^*M80T*^ was previously described to exhibit a modest phenotype at early time points [19], and these data corroborate our analyses of the GE *dTPI*^*M80T*^ allele (Fig 2B and 2C). Comparatively, the dimer interface mutants displayed a more severe degree of behavioral dysfunction than that seen in *dTPI*^*M80T*^ (Fig 2B and 2C). These data support the hypothesis that mutations at the dimer interface are sufficient to induce neurologic dysfunction.

Lysate isomerase activity was compared between samples taken from animals homozygous for the dimer interface mutants. First, it was noted that all dimer interface mutants exhibited reductions in TPI activity (Fig 2D). However, a comparison of the *dTPI*^*M80T*^, *dTPI*^*T73R*^, and *dTPI*^*G74E*^ lysates revealed a striking observation–the least phenotypically severe mutation (*dTPI*^*M80T*^) was characterized by the lowest isomerase activity (Fig 2D). These data support previous observations that TPI activity does not predict the presence or severity of TPI deficiency [25].

**Fig 3:**
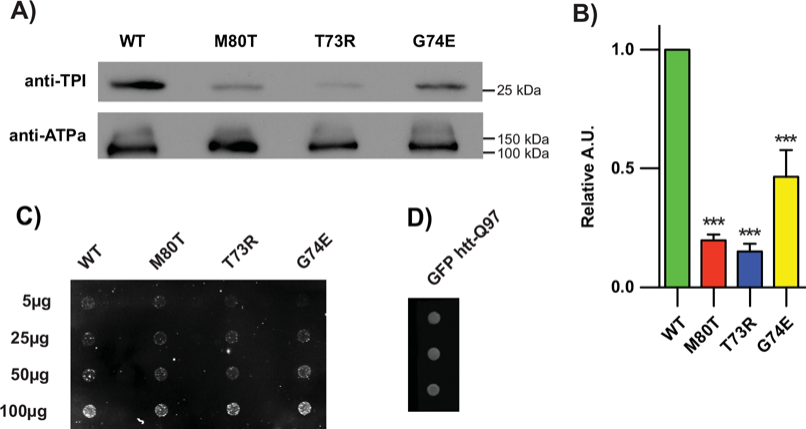
Mutations at the TPI dimer interface reduce TPI levels *in vivo* without aggregation. (A) *dTPI*^*T73R*^ and *dTPI*^*G74E*^ homozygote animal lysates display reduced protein levels with (B**)** quantification normalized to WT and an ATPalpha loading control, n = 3. (C) The reduction in SDS-soluble TPI is not caused by protein aggregation; increasing amounts of lysate were loaded and show no differences in filter-trapped TPI across all genotypes, (D) 10μg of huntingtin exon1-GFP lysate displayed robust retention on the filter, n = 2. Comparisons were made with a One-way ANOVA using Tukey’s post hoc test, *** p<0.001 relative to WT.

Many conformational diseases are elicited through changes in protein structure and stability leading to misfolding, then either sequestration and degradation, or aggregation [35]. First, we examined whether these new dimer interface alleles produced robust levels of TPI protein. We determined TPI levels in our dimer interface mutants as previously [34], and found that both *dTPI*^*T73R*^ and *dTPI*^*G74E*^ homozygotes exhibited reduced protein levels (Fig 3A and 3B).

It has previously been shown that TPI has the capacity to aggregate and thereby seed the aggregation of other proteins such as tau [31]. When measuring protein levels via SDS-PAGE, it is important to note that not all aggregate species are SDS soluble and a reduction in protein levels can indicate that the aggregates are not passing through the gel matrix. To determine whether the dTPI^T73R^ and dTPI^G74E^ proteins aggregate we used a dot-blot filter trap assay to assess retention differences between TPI mutant isoforms, as performed previously [36]. Lysates were collected from homozygous animals, and PC12 cell lysates expressing EGFP-huntingtin-Q97 (GFP htt-Q97) were used as a positive aggregation control. The results indicate that little TPI was trapped on the 200 nm filter, yet each sample showed a concentration-dependent increase in signal (Fig 3C). Importantly, no differences were observed in TPI signal between the WT and mutant alleles (Fig 3C). These data support similar findings established by sedimentation assays performed on *dTPI*^*M80T*^ [34], and led us to conclude that although these dimer interface mutants display reduced protein levels via SDS-PAGE, this is not due to the insolubility of large aggregates.

To date, all but one study examining TPI deficiency in *Drosophila* have highlighted a reduction in TPI protein levels in disease-associated alleles [18–20,25,26]. To independently examine the importance of TPI protein levels *in vivo*, we employed the GAL4-UAS expression system to knock down wild type (WT) TPI using a UAS-RNAi line directed toward *dTPI* messenger RNA (mRNA) [37]. These lines were driven with actin-GAL4 + UAS-GAL4 (actin/UAS-GAL4) to obtain a dramatic reduction of TPI in all tissues.

Using UAS-RNAi in conjunction with actin/UAS-GAL4, we found that *w;actin-GAL4*,*UAS-GAL4/+;UAS-RNAi*^*TPI*^*/+* animals exhibited a dramatic reduction in TPI protein levels similar to that seen in head and thorax tissue from *w;;dTPI*^*M80T*^ homozygotes (S3A and S3B Fig). Head and body tissues were assessed separately to ensure equivalent knockdown in both tissues. Next, we examined animal behavior in these knockdown populations to determine whether depletion of cellular TPI was sufficient to elicit TPI deficiency behavioral abnormalities. Mechanical stress responses were used to quantify behavioral dysfunction. None of the knockdown genotypes exhibited abnormal mechanical-stress dependent responses (S3C Fig). No paralysis or seizure-like activity was observed in the knockdown genotypes at elevated temperatures, though hypoactivity was noted, with the knockdown animals consistently dwelling near the bottom of the vial relative to their *TPI*^*+*^ and UAS only controls. These observations suggested that a general depletion of TPI is not sufficient to elicit paralysis or seizure-like locomotor dysfunction, yet do not exclude the possibility that changes in protein conformation, localized subcellular depletions, or changes in protein stability may play a greater role in animal pathology.

### A catalytically inactive TPI enzyme suppresses TPI deficiency

**Fig 4:**
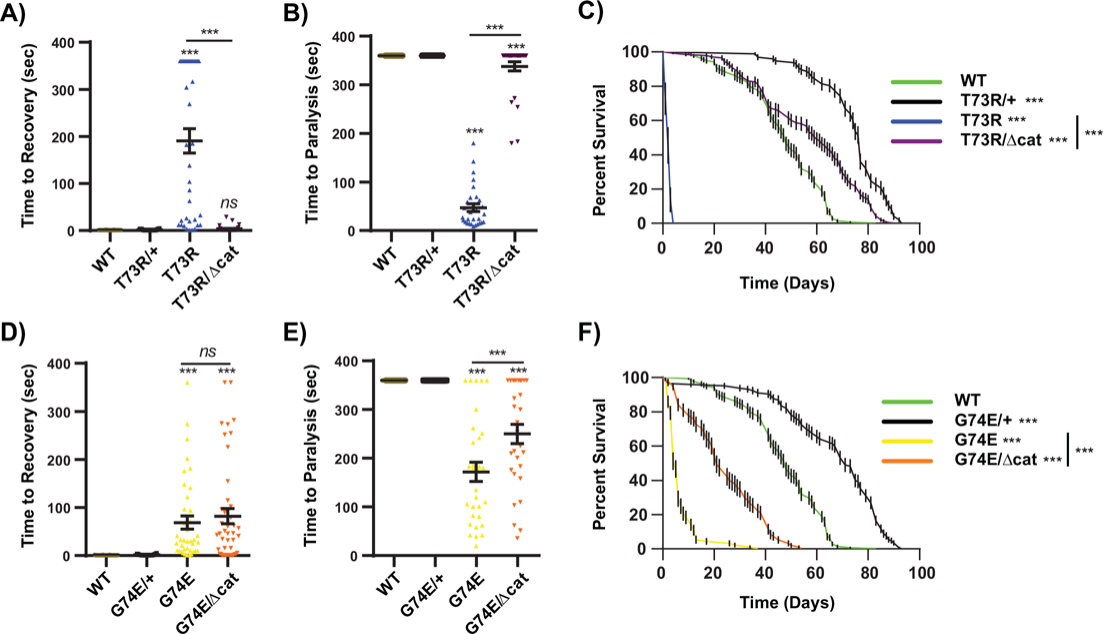
A catalytically inactive allele attenuates TPI dimer mutant behavior and longevity. (A) *dTPI*^*Δcat*^ complements *dTPI*^*T73R*^ mechanical and (B) thermal stress sensitivity and (C) longevity, and (D,E) partially attenuates behavior and (F) longevity defects of *dTPI*^*G74E*^. Thermal stress paralysis times at 360 sec. represent wild type behavior, the assay was stopped at 6 min. n≥30 for behavior, and n≥90 for lifespans. Behavior was compared using a One-way ANOVA with Tukey’s post hoc test, and lifespans by a Log-rank (Mantel-Cox) survival test, *ns* indicates no significance, and *** p<0.001.

Previous work demonstrated that a catalytically inactive allele of TPI (*dTPI*^*Δcat*^) complemented the behavior and longevity defects of the *dTPI*^*M80T*^ allele [25], a mutation now established to disrupt enzyme dimerization. This previous study suggested that TPI deficiency is a loss-of-function disease caused by either i) the depletion of cellular TPI, or ii) a conformational change that could be rescued through the addition of a properly folded yet catalytically open/inactive isoform [25]. Having utilized knockdown strategies to examine the necessity of total TPI levels, we sought to confirm the capacity of *dTPI*^*Δcat*^ (Lys-to-Met, position 11, Fig 1A) to complement additional dimer-interface mutations. To evaluate whether *dTPI*^*Δcat*^ was sufficient to support normal behavior and longevity, *dTPI*^*+*^*/dTPI*^*+*^, *dTPI*^*+*^*/dTPI*^*T73R*^, *dTPI*^*T73R*^*/dTPI*^*T73R*^, *dTPI*^*T73R*^*/dTPI*^*Δcat*^, *dTPI*^*+*^*/dTPI*^*G74E*^, *dTPI*^*G74E*^*/dTPI*^*G74E*^, and *dTPI*^*G74E*^*/dTPI*^*Δcat*^ animals were collected and tested as outlined above. These experiments demonstrated that the *dTPI*^*T73R*^ allele was nearly fully complemented by *dTPI*^*Δcat*^ (Fig 4A–4C), similar to the results found with *dTPI*^*M80T*^ [25]. It should be noted that this complementation was not fully penetrant; 5 out of the 30 *dTPI*^*T73R*^*/dTPI*^*Δcat*^ animals did paralyze after an extended thermal stress period (Fig 4B). The penetrance of the thermal stress complementation is reflected in an increased time to paralysis relative to the homozygous mutant animals (Fig 4B and 4E). *dTPI*^*G74E*^ was also complemented by *dTPI*^*Δcat*^, although more modestly than was observed for *dTPI*^*T73R*^. Mechanical stress responses were unchanged in *dTPI*^*G74E*^/*dTPI*^*Δcat*^ relative to *dTPI*^*G74E*^ homozygotes, though the penetrance of thermal stress sensitivity was decreased to 20 out of 30 animals, and the median lifespan of the *dTPI*^*G74E*^ mutants was extended from 5 to 21 days (Fig 4D–4F). Importantly, neither of the dimer interface mutants elicited dominant negative effects within the *dTPI*^*+*^ heterozygotes; to the contrary, *dTPI*^*T73R*^ and *dTPI*^*G74E*^ promoted a significant increase in animal health, extending the median 48 day *dTPI*^*+*^*/dTPI*^*+*^ lifespans to 77 and 71 days, respectively (Fig 4C and 4F). *In toto*, *dTPI*^*Δcat*^ partially but significantly complemented each of the new *TPI* dimer alleles.

### hTPI^Δcat^ crystal structure

**Fig 5:**
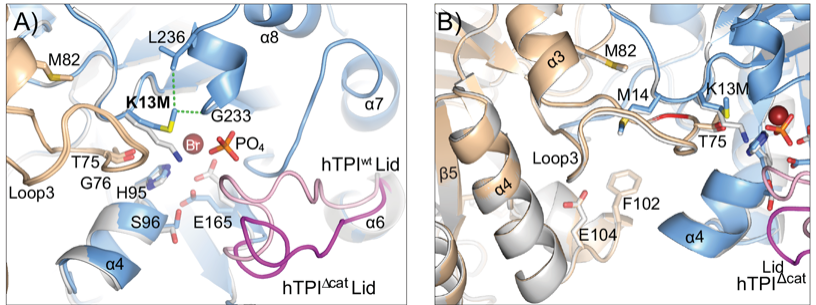
hTPI^Δcat^ crystal structure reveals a catalytically incompetent enzyme with an unaltered dimer interface. (A) The hTPI^Δcat^ adopts an open lid conformation. Superposition of hTPI^WT^ (grey) and hTPI^Δcat^ (blue) structures. Loop3 (tan), the position of the lid covering the TPI active site (magenta and pink), and the locations of bromide and phosphate ions from the active site pocket of hTPI^WT^ are indicated. New hydrophobic interactions that help to reposition M13 are shown (green). (B) The dimer interface of hTPI^Δcat^ is unchanged relative to hTPI^WT^. Superposition of hTPI^WT^ (grey) and hTPI^Δcat^ shown as in (A), with monomer subunits of hTPI^Δcat^ dimer in blue and tan. Key dimer interface residues and loops are indicated.

Our experiments with TPI dimer mutations demonstrated that alterations of the dimer interface were sufficient to elicit TPI neurologic dysfunction, and that these phenotypes were able to be complemented with *dTPI*^*Δcat*^. To address how the TPI^Δcat^ substitution may influence its structure, we purified, crystallized, and determined the structure of hTPI^Δcat^ at 1.7Å resolution, refining against native data to R_work_ and R_free_ values of 15.8%, and 19.6%, respectively (Table 1). These crystals grew in conditions that were nearly identical to conditions in which we have previously determined the structure of wild-type human TPI [18], minimizing the effects that changes in the crystallization condition or crystal packing might have on the resulting structure. While the overall fold of hTPI^Δcat^ is highly similar to wild-type (r.m.s.d of 0.35 Å over all atoms) there are a number of important differences within the catalytic pocket and neighboring regions. First, the active site pocket of our previous hTPI^WT^ structure contained a highly ordered phosphate and bromide ion located where the phosphate and triose groups of the natural substrate, DHAP, would be located [18]. In contrast, the active site pocket of hTPI^Δcat^ was filled with solvent. At the site of the hTPI^K13M^ substitution (hTPI^Δcat^), the M13 side chain adopts a different conformation than its lysine counterpart, shifting 4 Å away from the catalytic site and interacting with N11, G233 and L236 at the back of the pocket (Fig 5A). The sidechain positions of important active site residues S96 and E165 are also altered in hTPI^Δcat^, breaking critical solvent networks and shifting E165 2.7 Å away from the position it adopts in wild-type TPI [18] and substrate analog bound structures [38–40] (Fig 5A). Lastly, the lid moves as much as 7 Å away from the active site pocket, adopting an open conformation [40,41] (Fig 5A) and corroborating established kinetic data demonstrating that this enzyme is catalytically inactive [42]. These data are in agreement with a structure of yeast TPI^K12M,G15A^ containing two mutations within the active site [43], but were an important control to isolate the structural impact of hTPI^K13M^. Importantly, examinations of the dimer interface of hTPI^Δcat^ revealed that it is unchanged relative to hTPI^WT^ (Fig 5B). The peptide backbone and side chains of Loop3 form the majority of the TPI dimer interface, and as shown previously, perturbations of this loop disrupt TPI dimer stability [28–30,44]. The new crystal structure revealed that the backbone of Loop3 along with important side chains M14, T75, G76, M82, and E104, are unaltered in hTPI^Δcat^ (Fig 5B). These structural data indicated that TPI^Δcat^ homodimers are catalytically inactive, with no observable alterations of the overall folding of the monomers or their dimeric assembly (Fig 5B).

**Table 1:** Crystallographic Data collection and Refinement statistics.

	hTPI^ΔCAT^
**Data collection**	
Space group	P 2_1_2_1_2_1_
Wavelength (Å)	
Cell dimensions	
*a*, *b*, *c* (Å)	65.1, 73.5, 92.8
α, β, γ (°)	90.0, 90.0, 90.0
Unique Reflections	48,935
Resolution (Å)	50.0–1.70 (1.73–1.70)
*R*_merge_ (%)	6.3 (58.6)
*I* / σ*I*	42.0 (2.9)
Completeness (%)	100 (100)
Redundancy	12.3 (11.2)
**Refinement**	
Resolution (Å)	48.7–1.70 (1.73–1.70)
*R*_work_ / *R*_free_ (%)	15.8/19.6 (21.0/29.4)
FOM	0.87 (0.82)
Number of. atoms	
Protein	7,432
Other	16
Solvent	455
*B*-factors	
Protein	30.6
Other	54.1
Solvent	37.8
R.m.s. deviations	
Bond lengths (Å)	0.017
Bond angles (°)	1.25
Rotamer outliers (%)	1.81
Ramachandrian	
Outliers (%)	0.20
Allowed (%)	1.43
Favored (%)	98.4

*Values in parentheses are for highest-resolution shell.

### dTPI^Δcat^ forms heterodimers with mutant TPI subunits

Complementation of the *TPI* dimer mutant alleles with *dTPI*^*Δcat*^ suggested a physical and/or functional interaction between the two enzymes. Given the dimeric nature of TPI, we sought to first examine physical interactions between TPI species. All of the dimer-interface substitutions used in this study disrupt homodimerization (Figs 1 and S2), though no experiments had yet addressed how these alterations may change heterodimerization with dTPI^Δcat^. These putative heterodimers could support or inhibit a critical function of TPI.

**Fig 6:**
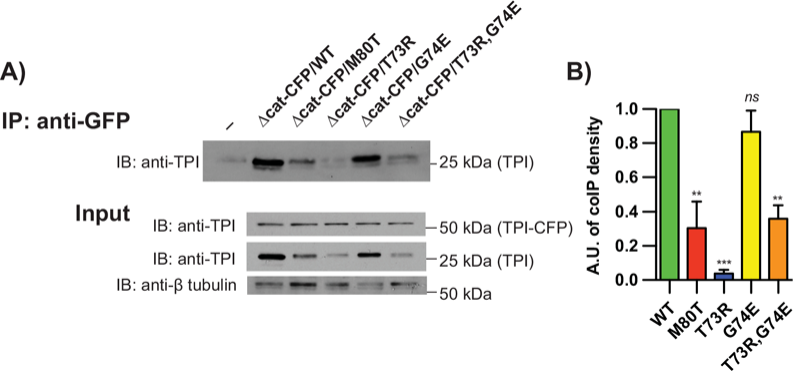
Heterodimerization of inactive TPI and dimer interface mutations. (A) dTPI^Δcat-CFP^ interacts modestly with dTPI^M80T^, dTPI^T73R^, and dTPI^T73R,G74E^, yet robustly with dTPI^G74E^. Representative immunoprecipitation and input blots are shown with (B) IP:anti-GFP quantification n = 3. Quantification represents 25kD TPI IP signal, with negative control subtracted, normalized to the lysate β-tubulin loading control, and compared to WT. Comparisons were made with a One-way ANOVA using Tukey’s post hoc test, *ns* indicates no significance, ** p<0.01, and *** p<0.001.

To examine heterodimer formation *in vivo*, we measured the capacity of the dimer-mutant TPI isoforms to co-precipitate using a C-terminal Cerulean cyan fluorescent protein (CFP) tagged variant of *dTPI*^*Δcat-CFP*^; this allele was previously confirmed to complement *dTPI*^*M80T*^ [25]. anti-GFP was covalently conjugated to the AminoLink resin, and the CFP tag was immunoprecipitated (IPed) in *dTPI*^*WT*^*/dTPI*^*Δcat-CFP*^, *dTPI*^*M80T*^*/dTPI*^*Δcat-CFP*^, *dTPI*^*T73R*^*/dTPI*^*Δcat-CFP*^, and *dTPI*^*G74E*^*/dTPI*^*Δcat-CFP*^ animal lysates and probed. Unconjugated resin was incubated with *dTPI*^*WT*^*/dTPI*^*Δcat-CFP*^ lysate and used as a negative control (-) (Fig 6A, IP). Upon elution and SDS-PAGE separation, protein size was used to discriminate between the tagged and untagged TPI isoforms; the CFP tag roughly doubled the molecular weight of dTPI-CFP monomer (~50 kDa) relative to dTPI monomer (~25kD) (Fig 6A, Input).

Robust amounts of dTPI^WT^ precipitated with dTPI^Δcat-CFP^, establishing substantial heterodimerization between the two species (Fig 6A and 6B) in agreement with the similarities between their respective dimer interfaces (Fig 5B). Conversely, dTPI^M80T^ and dTPI^T73R^ displayed markedly reduced associations with dTPI^Δcat-CFP^, corroborating their previously established dimerization deficiencies and reflecting their overall prevalence in the lysate (Fig 6A). Finally, it was surprising to see that dTPI^G74E^ produced heterodimerization similar to that seen in dTPI^WT^ (Fig 6A and 6B); it was predicted that the rotational flexibility of G74 was necessary for the appropriate positioning of loop 3 and establishment/rigidification of the dimer interface [28].

The coIP experiments suggested that the TPI species responsible for *dTPI*^*T73R*^ phenotype suppression in the animals was not a dTPI^Δcat^ heterodimer; the heterodimer was a very small fraction of the total TPI enzyme in lysate (Fig 6). Conversely, the dTPI^Δcat-CFP^::dTPI^G74E^ heterodimer existed as a substantial fraction of the total TPI (Fig 6), yet exhibited modest complementation of the abnormal behavioral phenotypes (Fig 3). The substantial and unanticipated presence of the dTPI^Δcat-CFP^::dTPI^G74E^ heterodimer could indicate an allele-specific dominant interaction. To examine whether we could enhance the capacity of *dTPI*^*Δcat*^ to suppress *dTPI*^*G74E*^, we designed a double-mutant aiming to revert heterodimer formation.

An allele was generated bearing both substitutions, *dTPI*^*T73R*,*G74E*^, and animals homozygous for this allele displayed aggressive behavioral phenotypes and shortened lifespans (S4A, S4B and S4D Fig). Immunoprecipitation experiments found that the double substitution reduced dTPI^T73R,G74E^ heterodimerization with dTPI^Δcat-CFP^ relative to dTPI^G74E^ (Fig 6). Finally, when paired with the *dTPI*^*Δcat*^ allele, the addition of the T73R substitution to *dTPI*^*G74E*^ enhanced the capacity for *dTPI*^*Δcat*^-behavioral complementation (S4A and S4B Fig). The mean time to recovery after mechanical stress was reduced from 204 sec. in *dTPI*^*T73R*,*G74E*^ homozygotes to 50 sec. in *dTPI*^*T73R*,*G74E*^*/dTPI*^*Δcat*^ animals with approximately 60% of the animals no longer responding to the stressor (defined as a recovery time ≤ 5 sec.) (S4A Fig); similar complementation was observed in the thermal stress assay (S4B Fig). Curiously, the longevity of the *dTPI*^*T73R*,*G74E*^*/dTPI*^*Δcat*^ animals was unchanged relative to *dTPI*^*T73R*,*G74E*^ homozygotes (S4D Fig); this is the second time that TPI deficiency behavioral abnormalities and longevity have not paralleled each other [18], suggesting the possibility of independent pathogenic mechanisms (see Discussion).

The inverse correlation between dTPI^Δcat^ heterodimerization and behavioral complementation suggested that *dTPI*^*Δcat*^ did not complement TPI deficiency behavioral phenotypes via heterodimer formation. Further, disease severity did not correlate with isomerase activity (S5B Fig); complementation of the dimer-mutant alleles with *dTPI*^*Δcat*^ failed to increase isomerase activity and in all but one case significantly decreased activity (S4C and S5 Figs). These data led us to conclude that *dTPI*^*Δcat*^ does not “suppress” TPI deficiency behavioral phenotypes through a general influence on TPI catalytic activity.

### Severe TPI dimer impairment alters synaptic function

TPI deficiency complementation was not corroborated by an enhancement of TPI catalysis; however, the mean temperature-dependent time to paralysis of the *dTPI*^*T73R*^ allele (27 sec) was a striking result (Fig 2B) and suggested a previously unknown role of TPI. Rapid (<60 sec.) temperature-dependent paralysis had only been identified in a handful of mutants in *Drosophila* and typically results from neural conductance or synaptic vesicle recycling defects [45]. To determine whether TPI was influencing vesicle dynamics, we first examined vesicle endocytosis at the synapse using the lipophilic dye, FM1-43. FM1-43 is a water soluble membrane dye that increases its fluorescence when bound to cellular membranes. During endocytosis, the dye will bind to the outer leaflet of the plasma membrane and become internalized within the synapse providing an optical measurement of endocytosis. Measuring vesicle dynamics in this context allowed us to assess two possibilities; i) a primary recycling defect due to impaired endocytosis, or ii) a secondary recycling defect due to aberrant exocytosis.

**Fig 7:**
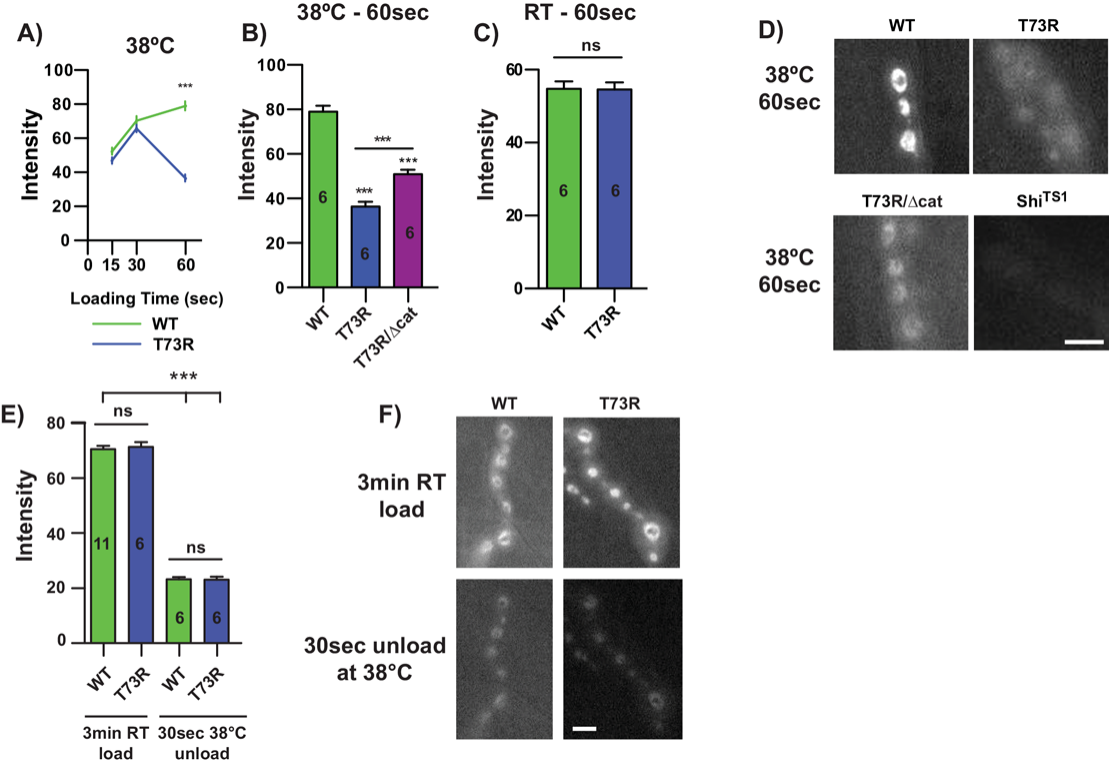
*dTPI*^*T73R*^ impairs NMJ synaptic vesicle dynamics. (A) An FM1-43 timecourse at the NMJ with loading times of 15, 30, and 60 sec., (B) with quantification of 60 sec. at 38°C, and (C) 60 sec. at room temperature (RT). (D) Representative images of *dTPI*^*WT*^, *dTPI*^*T73R*^, *dTPI*^*T73R/Δcat*^ and *Shi*^*ts1*^, n = 6. (E) FM1-43 unloading is unchanged between *dTPI*^*WT*^ and *dTPI*^*T73R*^ at 38°C with animal replicates indicated, and (F) representative images. Comparisons were made with a One-way ANOVA using Tukey’s post hoc test, ***p<0.001. Scale bars = 5μm.

We dissected larvae homozygous for *dTPI*^*WT*^, *dTPI*^*T73R*^, and *Shi*^*ts1*^ as previously detailed [46]. The NMJ preparations were heated to 38°C over 3 min. and a loading curve was generated from a series of three different high [K^+^] + FM1-43 loading times– 15 sec., 30 sec., and 60 sec. as previously detailed [47]. *dTPI*^*WT*^ displays a progressive increase in dye loading from 15 sec. to 60 sec. (Fig 7A), while the temperature sensitive dynamin mutant control *Shi*^*ts1*^ showed no signs of vesicle recycling at any heated time points (Fig 7D, data not quantified). Conversely, although *dTPI*^*T73R*^ displayed similar loading to *dTPI*^*WT*^ at 15 and 30 sec., *dTPI*^*T73R*^ exhibited a striking 50% decrease in loading at 60 sec. (Fig 7A, 7B and 7D). This progressive decrease in endocytosis was stimulation and temperature dependent; loading experiments performed at room temperature did not exhibit an endocytic defect (Fig 7C). As previous experiments had highlighted the capacity of *dTPI*^*Δcat*^ to complement the adult behavioral defects of *dTPI*^*T73R*^, we examined *dTPI*^*T73R*^*/dTPI*^*Δcat*^ larvae to assess the relationship between vesicle endocytosis and animal behavior. The *dTPI*^*T73R*^*/dTPI*^*Δcat*^ animals displayed a significant increase in vesicle endocytosis relative to *dTPI*^*T73R*^ (Fig 7B and 7D). These results demonstrate that *dTPI*^*Δcat*^ complements adult behavior and vesicle endocytosis defects. The utilization of chemical stimulation in these preparations demonstrates a synaptic defect arising from the severe *dTPI*^*T73R*^ dimer mutation as this methodology bypasses conductance requirements.

A reduction in vesicle dye uptake could be derived from defects in endocytosis or exocytosis, and indeed, these activities are intimately linked [48]. To examine temperature-dependent changes in vesicle fusion, *dTPI*^*WT*^ and *dTPI*^*T73R*^ animals were i) loaded with dye at RT for 3 min., ii) washed with 0 mM Ca^2+^ HL-3, iii) imaged, iv) heated to 38°C, v) vesicle fusion initiated with 30 sec. of high [K^+^] HL-3, and vi) reimaged. Care was taken to ensure the same synapses were imaged at loading and unloading timepoints. Preliminary experiments demonstrated that 60 sec. of high [K^+^] stimulation completely unloaded the synapses in each genotype; therefore 30 sec. was analyzed to achieve a measurable dynamic range. Unloading experiments at elevated temperatures demonstrated no change in vesicle exocytosis between *dTPI*^*WT*^ and *dTPI*^*T73R*^ at 38°C (Fig 7E and 7F).

**Fig 8:**
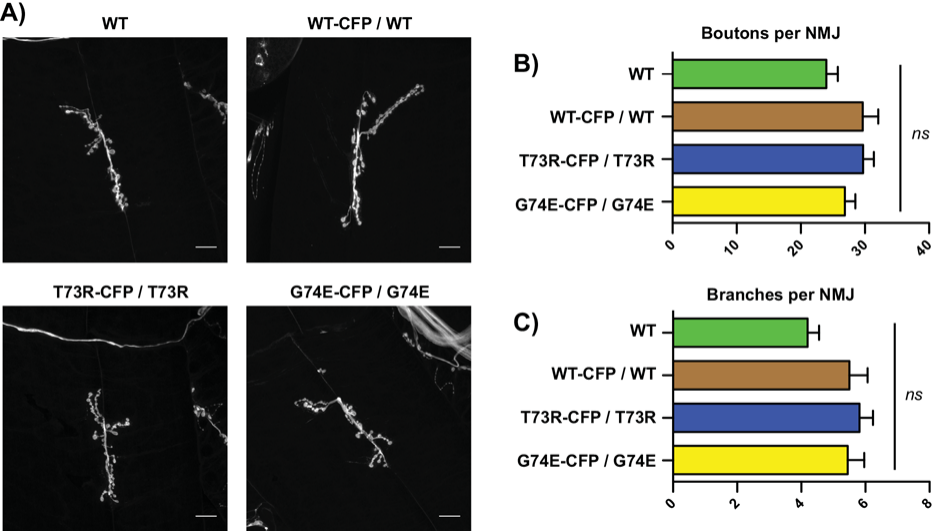
TPI dimer interface substitutions do not alter NMJ development and morphology. (A) NMJ morphology of segment A2 muscle 6/7 was characterized for (B) bouton number and (C) branching. Boutons were defined as varicosities greater than 2 μm in diameter. Neither parameter showed significant differences elicited by the mutations, relative to either *dTPI*^*WT*^ or *dTPI*^*WT-CFP*^*/TPI*^*WT*^. CFP tags did not alter the behavioral deficits of the animals (S6 Fig). n = 10. Comparisons were made with a One-way ANOVA using Tukey’s post hoc test, *ns* indicated no significance. Scale bar = 10 μm.

Finally, functional changes at the synapse can be the result of acute impairments in recycling machinery or more chronic developmental defects. Mutations in the E3- ubiquitin ligase *Highwire* or alterations in the *trans-*synaptic signaling proteins *wingless* and *Glass-bottom boat* have been shown to alter synaptic function through primary changes in development [49–51]. These changes in synaptic physiology are accompanied by dramatic alterations in synaptic morphology, a hallmark of neurodevelopmental defects. To examine whether aberrant neurodevelopment may contribute to this recycling deficit, we morphologically characterized the *Drosophila* NMJ from segment A2, muscle 6/7; this particular NMJ is highly elaborate and therefore sensitive to developmental perturbations. The *dTPI*^*M80T*^, *dTPI*^*T73R*^, and *dTPI*^*G74E*^ alleles all exhibited early lethality if maintained at 25°C, therefore development was scored at RT. Third instar larva were dissected, and an assessment of bouton number and branches revealed no significant developmental differences in the thermal-stress sensitive mutants relative to *dTPI*^*WT*^ (Fig 8). These results suggest that the synaptic defect is an acute disruption of function, and not likely a secondary defect caused by altered development. Collectively, these data demonstrate that TPI deficiency thermal-stress sensitivity is characterized by acute perturbation of synaptic vesicle dynamics.

## Discussion

We conclude that the impairment of TPI dimerization is sufficient to elicit neurologic dysfunction. Dimerization of TPI is critical to its conformation, stability, and catalytic properties; and disruption of these molecular properties impedes vesicle dynamics at the synapse. Impaired synaptic function is thermal-stress dependent, and both vesicular and behavioral abnormalities can be genetically complemented through a catalytically inactive *TPI* allele.

### Triosephosphate isomerase dimerization

The pathogenic hTPI^M80T^ substitution impairs TPI dimerization. These results were obtained from purified proteins and do not corroborate those from non-denaturing gel filtration experiments performed on animal lysates [26]. However, several *in vitro* studies have found that mutations that impair TPI dimerization severely destabilize the protein [28,30,44,52,53]. *In vivo*, unstable proteins are bound by chaperones and either refolded, targeted to the proteasome, or aggregate [54]. The results presented here suggest that dTPI^M80T^ does not cause TPI to aggregate (Fig 3), while previous work extensively details the recruitment of Hsp70 and Hsp90 to dTPI^M80T^ and its degradation through the proteasome [34]. Therefore, we hypothesize that TPI monomer may not have been detected previously in animal lysates due to its rapid sequestration and degradation. We believe these data, along with the previous inability to identify monomer *in vivo*, collectively suggest that TPI does not stably exist *in vivo* as a soluble monomer.

We utilized our GE system to generate two additional *TPI* alleles with point mutations at the dimer interface that have previously been shown to impair homodimerization [28]. These substitutions were located at the tip of the 3^rd^ loop of TPI that extends into its dimer partner and stabilizes/rigidifies a network of hydrophobic interactions and hydrogen bonds which form the dimer interface [28,29,55]. The substitution of these dimer interface residues resulted in severely pathogenic *TPI* alleles, eliciting greater behavioral dysfunction and shorter lifespans than *dTPI*^*M80T*^ (Fig 2).

### Molecular basis of TPI deficiency

A universal molecular mechanism of TPI deficiency pathogenesis is currently unclear. To date, two crystal structures of disease-associated TPI mutations have been reported [15,18]. These mutations were found in two distinct structural regions of the TPI homodimer. The first structure to be solved was from the most commonly diagnosed TPI deficiency substitution, hTPI^E104D^ [15]. The hTPI^E104D^ substitution is a conservative alteration of a charged residue at the dimer interface that results in reduced dimer stability, but unchanged catalytic activity [15,16]. The second disease-associated structure was an hTPI^I170V^ substitution, a conservative substitution found on the catalytic lid of the enzyme that enhances thermal stability and reduces catalytic activity [16,18]. The dimer substitutions used in this study share several molecular characteristics with the TPI^E104D^ human mutation, and given the conservation of the TPI enzyme, we propose that these patients likely share similar molecular and cellular dysfunction as identified in this study. Conversely, the dimer substitutions are not predicted to share many molecular similarities with the hTPI^I170V^ mutation indicating that dimerization defects are sufficient but not necessary to elicit TPI deficiency. It is interesting to note that although *dTPI*^*T73R*^ and *hTPI*^*I170V*^ both exhibit mechanical and thermal stress sensitivities in *Drosophila*, the behavioral dysfunction caused by the dimer substitutions is far more severe, and *hTPI*^*I170V*^ does not influence animal longevity [18]. Further, the capacity to attenuate behavioral dysfunction but not longevity in *dTPI*^*T73R*,*G74E*^/*dTPI*^*Δcat*^ suggests an independent pathogenic mechanism (S4 Fig) that may not be determined by TPI dimerization.

TPI dimerization and protein stability *in vivo* will ultimately influence catalytic capacity, and many TPI activity measurements from animal models and patient tissue samples have identified a reduction in isomerase activity [7,14,17,18,25,56–58]. However, our measurements of isomerase activity from healthy and affected animal lysates argue that TPI dimer integrity is a stronger determinant of behavioral dysfunction. Still, this could indicate that reduced TPI activity is corollary to the disease or a contributing factor to an alternative pathogenic mechanism. Previous studies have demonstrated that the redox state is altered in TPI deficient cells and organisms [33,59,60]. The redox status in TPI deficiency is proposed to be altered by abnormal flux through the pentose-phosphate pathway as well as potential accumulation of advanced glycation end-products (AGEs) [16,20,33,59–61], and the accumulation of redox damage in the nervous system is strongly linked with several neurodegenerative diseases [62–64]. Interestingly, a study in yeast demonstrated differences in redox responses between the E104D and I170V mutations [16]; these results could imply different modes of pathogenesis that are dependent on the conformational and catalytic states of TPI.

### hTPI^Δcat^ predicted heterodimers

One unresolved aspect of this study was the inability of *dTPI*^*Δcat*^ to fully complement the behavioral defects of *dTPI*^*G74E*^. Co-IP experiments suggested that complementation correlated with an inability of dimer-interface mutants to form heterodimers with dTPI^Δcat^ (Fig 6). These data would imply that dTPI^G74E^ may be exhibiting a dominant negative effect as a heterodimer, but this conclusion was inconsistent with our genetic analyses of *dTPI*^*WT*^/*dTPI*^*G74E*^ animals (Fig 4). To investigate whether TPI^G74E^ may be interacting differently with TPI^WT^ than with TPI^Δcat^, we purified, crystallized, and determined the structure of hTPI^Δcat^ at 1.7Å resolution.

**Fig 9:**
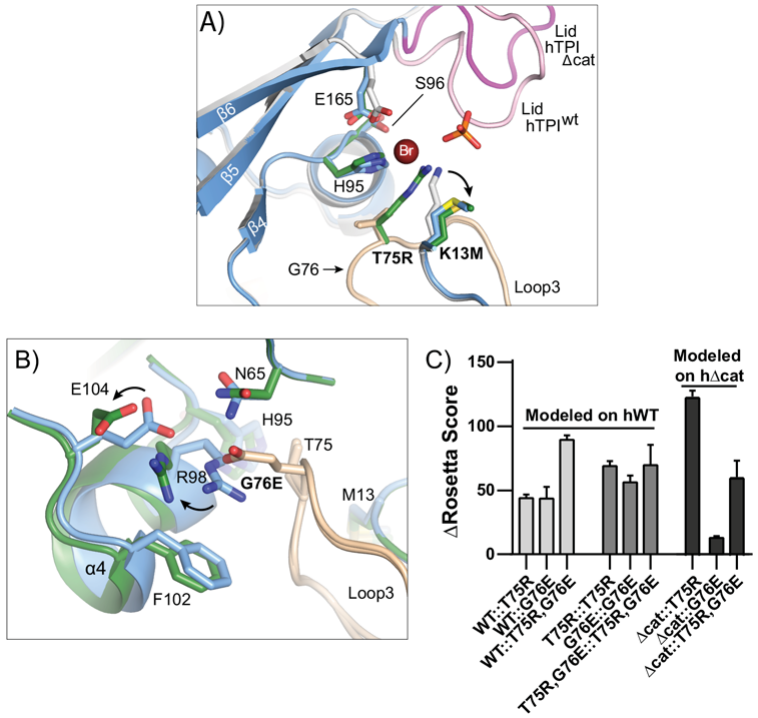
hTPI^Δcat^ models predict that hTPI^Δcat^::hTPI^G76E^ heterodimerization alters the TPI dimer interface. (A) The R75 side chain may insert itself into the active site in the context of a hTPI^Δcat^::hTPI^T75R^ heterodimer. Shown is a superposition of hTPI^WT^ (grey), hTPI^Δcat^ (blue), and hTPI^Δcat^::hTPI^T75R^ (green) obtained from Rosetta Backrub modeling. Loop3, containing T75R and G76E, is indicated in tan. For clarity, the mainchain of hTPI^Δcat^::hTPI^T75R^ has been omitted. (B) Repositioning of E104 and R98 side chains assists the dimer interface to accommodate the G76E substitution in Rosetta modeling. Superposition of hTPI^Δcat^ structure (blue) with the hTPI^Δcat^::hTPI^G76E^ model (green). In both structures, Loop 3 is shown in tan and selected active site pocket residues are indicated. (C) Modeling the effect of T75R and G76E substitutions in the context of hTPI^WT^ and hTPI^Δcat^ structures. Normalized scores for the best 10% of 50 simulations were averaged for each experimental structure with the indicated computational substitution. Higher scores indicate a resulting model that is less favorable using the Rosetta energy function.

While the hTPI^K13M^ substitution (hTPI^Δcat^) resulted in multiple rearrangements that mimic the open or non-catalytic TPI conformation, the dimeric interface remained essentially unchanged, including the peptide backbone of Loop3 and sidechain positions hT75, hG76, and hM82 (Fig 5). To address how hTPI^T75R^ and hTPI^G76E^ substitutions may influence the dimer interface, we generated models of heterodimers in which one subunit contained either hT75R, hG76E, or both hT75R and hG76E substitutions, while the other monomer remained unaltered. Models were made using either wild-type TPI hTPI^WT^ (PDB: 4POC) or the hTPI^Δcat^ (PDB: 4ZVJ) as the structural template, and subjected to analysis by RosettaBackrub [65]. Briefly, Rosetta scores are predictions of the most energetically stable conformations with higher scores indicating less favorable positioning of the model. The algorithm was run 50 times for each mutation to be modeled. Of these 50 simulations, the lowest scores of hTPI^Δcat^::hTPI^T75R^ and hTPI^Δcat^::hTPI^G76E^ were selected and shown (Fig 9A and 9B), while the modeled structures whose Rosetta scores fell within the best 10% of its respective ensemble were collected for analysis (Fig 9C).

Modeling the hTPI^T75R^ and hTPI^G76E^ substitutions as homodimers or heterodimers with the hTPI^WT^ structure produced high Rosetta scores, predicting poor energetic favorability (Fig 9C) in agreement with our gel filtration experiments (S2 Fig). To examine TPI^Δcat^ heterodimers we used the new hTPI^Δcat^ structure to model hTPI^Δcat^::hTPI^T75R^ and hTPI^Δcat^::hTPI^G76E^. These experiments predicted a high Rosetta score for hTPI^Δcat^::hTPI^T75R^ and a very low one for hTPI^Δcat^::hTPI^G76E^, corroborating the results of our animal lysate coIP experiments and suggesting the simulated heterodimers may accurately represent the conformations of these molecules.

The hTPI^Δcat^::hTPI^T75R^ heterodimer with the lowest Rosetta score suggests the hR75 residue may orient toward the catalytic pocket of hTPI^Δcat^, lining the floor of the substrate-binding pocket through the displacement of hE165 and hK13M (Fig 9A). This orientation of hR75 into the catalytic pocket is similar to that previously described by Wierenga and colleagues [28]. The hTPI^Δcat^::hTPI^G76E^ heterodimer with the lowest Rosetta score suggests that hE76 finds a stable position in the dimer interface through the displacement of hE104 and hR98, possibly via coordination of the terminal amide of hN65 (Fig 9B). Interestingly, perturbation of hE104 has been shown to significantly alter the TPI dimer interface and elicits TPI deficiency in humans through a conservative hTPI^E104D^ substitution [15]. These modeling predictions suggest that the character of the hTPI^Δcat^::hTPI^G76E^ dimer interface is drastically altered relative to hTPI^Δcat^ homodimers.

### Triosephosphate isomerase deficiency neuropathogenesis

*Drosophila* TPI deficiency neurologic dysfunction is characterized by impaired vesicle dynamics at the neuronal synapse, a defect we believe is likely conserved in human patients. The key to deciphering this pathogenic mechanism was the behavioral severity of the newly generated TPI dimer interface mutants. The *dTPI*^*T73R*^ allele exhibited temperature-dependent paralysis at a mean time of approximately 27 sec., an acute behavior that is rare and highly enriched for synaptic or conductance defective mutants. Only a handful of *Drosophila* mutant alleles have been identified with rapid temperature-dependent paralysis, including those of voltage-gated Na^+^, K^+^, and Ca^2+^ channels (*para*, *sei*, *cac*) [66–68], the sodium-potassium exchanging ATPase (*ATPα*) [69], and components of vesicle fusion and recycling (N-ethylmaleimide sensitive factor–*dNSF1*, dynamin–*Shi*) [70,71]; and after noting these phenotypic similarities we broadly examined synaptic function. Stimulation was conducted using high [K^+^] bath applications, thereby bypassing the participation of Na^+^ and K^+^ channels. The *dTPI*^*T73R*^ mutants were characterized by normal endocytosis during acute stimulations (15 and 30 sec.) but exhibited a dramatic reduction after 60 sec. of stimulation (Fig 7A), suggesting a time/excitation dependent phenotype. Further, complementation with the *dTPI*^*Δcat*^ allele significantly increased the temperature-dependent FM1-43 loading at these terminals (Fig 7B and 7D), similar to its complementation of adult thermal stress-induced paralysis. Finally, measurements of vesicle exocytosis at these elevated temperatures did not indicate an exocytic defect (Fig 7E and 7F). We were unable to detect terminal signal above background after 60 sec. of unloading, so we cannot unequivocally define the nature of the vesicular dysfunction, but the data suggest it is likely due to impaired endocytosis.

The observed synaptic defect provides insight into why the majority of human patients present with TPI mutations affecting the dimer interface. First, all substitutions that disrupt the dimer interface have been shown to destabilize the enzyme *in vitro* [28,29,55]. This destabilization is likely responsible for the reduced cellular TPI found in patient samples, and our work with the *dTPI*^*M80T*^, *dTPI*^*T73R*^, and *dTPI*^*G74E*^ mutants provide additional evidence that dimer interface substitutions reduce TPI levels *in vivo* (Fig 3A and 3B). Secondly, the cellular depletion of dTPI^M80T^ has been shown to be mediated by heat shock protein sequestration and proteasomal degradation [34]. If chaperones sequestered and degraded these misfolded or unstable proteins, this would likely prevent the distribution and maintenance of TPI at specific subcellular locales. Recent work has shown that the anterograde transport of globular/soluble proteins to the terminals is a slow process, moving at a rate of approximately 0.008–0.01μm/sec [72]. To put this in the context of the *Drosophila* nervous system, the length of the relatively short larval motor axon innervating muscle 4 of segment A3 has been measured to be ~220μm [73]. Based on these approximations, one could estimate that it would take ~6 hrs for TPI translated in the soma to be transported to the axonal terminal. In this way, substitutions that affect protein stability would likely result in improper localization or sequestration of TPI during distal transport, ultimately depleting TPI at the synapse. This proposal is also consistent with the inability of RNAi knockdown to recapitulate *Drosophila* TPI deficiency behavioral phenotypes, as RNAi alters mRNA transcript levels rather than enzyme conformation or stability. RNAi knockdown of TPI^WT^ would reduce, but still allow the transport and stable accumulation of TPI^WT^ at the synaptic terminal.

Two other *Drosophila* glycolytic mutants, aldolase and phosphoglycerate kinase, have been shown to exhibit temperature-dependent paralysis though with longer onsets [74,75]. The phosphoglycerate kinase mutant was shown to exhibit synaptic dysfunction, and the authors asserted that an inhibition of vesicle recycling was likely the cause of the functional defect [75]. In both cases, the animals were found to have depletions in lysate ATP [74,75]. It is attractive to speculate that all three of these glycolytic mutants may suggest a pivotal role for glycolysis within synaptic vesicular dynamics, and indeed, recent measurements of ATP consumption in the synapse suggest that glycolytic ATP is the primary substrate used to support synaptic function [76,77]. However, the role of glycolytic proteins and their putative energetic importance at the synapse is controversial. Many research groups assert the preeminent utilization of mitochondrial ATP at these sites [78–81], while the lactate shuttle hypothesis largely circumvents a role for neuronal glycolysis [82,83]. Further, the absence of a correlation between TPI catalytic activity and behavioral phenotypes suggests that the enzyme may be complexing with another molecule to facilitate synaptic vesicle cycling.

How the dimerization or integrity of the TPI dimer interface impacts synaptic vesicle dynamics remains a mystery, though one candidate for a molecular complex is the actin-regulatory protein cofilin. In *Drosophila*, cofilin (*twinstar)* and *twinfilin* mutants have been demonstrated to elicit functional and developmental neurologic defects [84–86], and actin-regulatory proteins such as cofilin, actin-depolymerizing protein (ADP), and twinfilin are known to influence synaptic vesicle dynamics [85,87,88]. Recently, cofilin was found to bind to TPI in both its inactive and active forms [89]. The precise binding site between cofilin and TPI is unknown, though with its mixture of charged and hydrophobic pockets, the TPI dimer interface may provide a suitable site for this interaction. Additional studies will be needed to specifically delineate the role of TPI in the synapse.

In conclusion, to our knowledge this work is the first to highlight a critical role for TPI in the cycling of vesicles at the synapse, with behavioral correlates similar to the inactivation of vesicle fusion/recycling proteins. These observations help clarify the neurologic symptoms seen in patients and will direct future therapeutic strategies. The findings of this study will guide future investigations regarding the contribution of TPI localization and function to synaptic vesicle dynamics, and ultimately how these properties are perturbed in TPI deficiency.

## Materials and Methods

### Animal strains

The Vienna *Drosophila* RNAi Center (VDRC) line used for knockdown experiments was stock #25644 [37]; experiments were also conducted with #25643 with similar results. The *w;actin-GAL4*,*UAS-GAL4;* animals were generated by recombining the second chromosomes of the *Drosophila* Genetic Resource Center (DGRC) stock *y*^*1*^ *w*^*1118*^*; P(w*^*+mC*^ *= UAS-Gal4*.*H)12B* and Bloomington Stock Center stock *y*^*1*^w*; P(Act5C-GAL4)25FO1/CyO,*y*^*+*^; recombinants were screened molecularly and balanced in a *w*^*1118*^ background. The following TPI alleles in this study were generated using the GE system: *dTPI*^*WT*^, *hTPI*^*WT*^, *dTPI*^*WT-CFP*^, *dTPI*^*M80T*^, *hTPI*^*M82T*^, *dTPI*^*T73R*^, *dTPI*^*T73R-CFP*^, *dTPI*^*G74E*^, *dTPI*^*G74E-CFP*^, *dTPI*^*T73R*,*G74E*^, *dTPI*^*Δcat*^, and *dTPI*^*Δcat-CFP*^. The development of the GE system and the production of the *dTPI*^*WT*^, *dTPI*^*M80T*^, *dTPI*^*Δcat*^, and *dTPI*^*Δcat-CFP*^ alleles were initially described elsewhere [25]. Briefly, GE involves the replacement of the TPI gene locus with a phiC31 integration site through homologous recombination. The phiC31 integration system allows nearly seamless integration of complementary vector constructs directly into the TPI gene locus to maintain endogenous spatial and temporal regulation. The *TPI*^*null*^ allele used in S1 Fig was generated previously, formerly known as *TPI*^*JS10*^ [19]. RNAi experiments used the *TPI*^*M80T*^ allele formerly known as *TPI*^*sgk*^ [19], due to similarities with the UAS-RNAi and GAL4 genetic backgrounds.

This study uses the established nomenclature for TPI, assuming the start methionine is removed following translation [13]; all residue numbering in this study uses the same convention. An alignment is included for clarification (Fig 1A). All animal populations assessed were approximately equivalent mixtures of males and females.

### Mutagenesis and genomic engineering

Site directed mutagenesis was performed using the QuikChange Lightening Site-Directed Mutagenesis Kit (Agilent Technologies). Mutagenesis primers were generated (Integrated DNA Technologies) to introduce a Thr-to-Arg codon change at position 73, and a Gly-to-Glu change at position 74 –both separately and together for the purpose of creating the double-mutant. Mutagenesis was performed on the previously published *pGE-attBTPI*^*+*^ plasmid [25] and confirmed by sequencing. Once the constructs were generated, *TPI* GE was performed using previously published methods [25,90,91]. Briefly, the *PGX-TPI* founder animals were mated to *vasa-phiC31*^*ZH-2A*^ animals expressing the integrase on the X chromosome and their progeny injected with *pGE-attBTPI* constructs. Integration events were identified via the *w*^*+*^ phenotype and verified molecularly.

### Human TPI enzyme purification

Human TPI enzyme was purified as outlined previously [18].

### Dynamic Light Scattering

DLS measurements were taken using a DynaPro Plate reader (Wyatt Technology) equipped with a temperature control unit. Purified BSA (Sigma Aldrich), hTPI^+^, and hTPI^M80T^ were diluted to concentrations of 3.75 μM, 15 μM and 30 μM in 100 mM triethanolamine (TEA); pH 7.6. Three 75 μl aliquots were loaded onto a 384-well microplate and read at 37°C. Ten measurements were taken per sample and Dynamics V6 software (Wyatt Technology) was used to process the scattering data, generating autocorrelation functions. Autocorrelation functions were then analyzed to obtain the hydrodynamic radii. Student’s T test was used to compare samples. DLS experiments were performed three times.

### Gel filtration chromatography

Gel filtration was performed as outlined previously [26]. Purified TPI samples were diluted to 15 μM in mobile phase, 100 μl were injected in triplicate, and their elution monitored at 280 nm. Experiments were performed three times. Chromatography traces were collected and analyzed using EZStart 7.3 (Shimadzu) to quantify the relative monomer and dimer populations. Curve integration data were compared using Student’s T test.

### TPI enzyme assays

Isomerase activity was determined using an NADH-linked assay as previously detailed [25,92]. Lysates were diluted to 0.1 μg/μl in 100 mM TEA pH 7.6 + inhibitors and enzyme activity was assessed. Reaction assays were performed in triplicate using 80 μl mixtures composed of 0.5 mM NADH, 0.752 mM GAP, 1 unit glycerol-3-phosphate dehydrogenase and 1 μg of lysate protein in 100 mM TEA; pH 7.6. Consumption of NADH was monitored at 340 nm and 25°C using a SpectraMax Plus 384 microplate reader (Molecular Devices). All reactions were performed at least three times. Reaction components were purchased from Sigma-Aldrich. Enzyme activity curves were normalized to reactions performed without GAP. A one-way ANOVA was performed to assess variance, and data sets were compared using Tukey’s post-hoc analysis.

### Behavioral testing and lifespan analysis

Mechanical stress sensitivity was examined on Day 1 by vortexing the animals in a standard media vial for 20 seconds and measuring time to recovery, similar to [93]. Briefly, recovery is defined as two purposeful WT movements including righting, grooming, climbing, or walking. Thermal stress sensitivity was assessed on Day 2 by acutely shifting animals to 38°C and measuring time to paralysis, as previously described [24,69]. In these assays, the animals typically seized and either flipped over onto their backs or fell sideways with no successive coordinated movements, i.e. righting, climbing, walking, grooming. Behavioral responses were capped at 360 and 600 seconds where indicated and reported as 360 and 600 sec. Animal lifespan determinations were performed at 25°C as previously described [69]. All assays used approximately equivalent numbers of males and females. One-way ANOVAs were performed with Tukey's post-hoc analysis to compare behavior, and lifespans were assessed with Log-rank (Mantel–Cox) survival tests.

### Immunoblots

Animals were collected and aged 1–2 days at room temperature. Ten fly heads were obtained in triplicate from each genotype and processed as described previously [34]. Blots were incubated with anti-TPI (1:5000; rabbit polyclonal FL-249; Santa Cruz Biotechnology), anti-ATPalpha (1:10,000; mouse monoclonal alpha5; Developmental Studies Hybridoma Bank), or anti-β tubulin (1: 6,000; rabbit polyclonal H-235; Santa Cruz Biotechnology). Densitometric analyses of the scanned films were performed on unsaturated exposures using ImageJ software available from the National Institutes of Health. A one-way ANOVA was performed to assess variance of TPI levels and data sets were compared using Tukey's post-hoc analysis.

### Filter-trap dot blot

The filter-trap dot blot was modified from methods published previously [36]. Animals were aged 1–2 days, collected and homogenized in 1X PBS (2.7 mM KCl, 137 mM NaCl, 2 mM NaH_2_PO_4_, 10 mM Na_2_HPO_4_; pH 7.4) supplemented with cOmplete mini Protease Inhibitors (Roche Diagnostics), and diluted to 1 μg/μl; wells were loaded as indicated. Samples were diluted 1:2 in 1% SDS, 1X PBS, boiled for 5 min., and filtered through a cellulose acetate membrane (Whatman, 0.2μm pore) using a 96-well vacuum dot blot apparatus. Positive controls were collected from PC12 cells transfected with a construct expressing huntingtin “exon1” bearing a stretch of 97 glutamines and C-terminally tagged with EGFP. The membrane was washed four times with 1% SDS-PBS, blocked with Odyssey Blocking Buffer (LiCor), and primary antibodies applied in Odyssey Blocking Buffer. Blots were incubated with anti-TPI (1: 5000) and anti-GFP (1: 5000; rabbit polyclonal; FL; Santa Cruz Biotechnology). The membranes were then washed and incubated with the secondary antibody IRDye 800-conjugated goat anti-rabbit (LiCor) at 1: 20,000 in Odyssey Blocking Buffer. Direct-to-scanner detection and blot visualization were performed using a LiCor Odyssey scanner. Filter-trap experiments were performed twice.

### Coimmunoprecipitation experiments

Coimmunoprecipitation was performed using the Pierce Co-Immunoprecipitation Kit (Thermo Scientific) as per manufacturer’s instructions. Lysates were generated by mechanically homogenizing 50 animals in 0.5 ml of IP Lysis buffer (25 mM Tris, 150 mM NaCl, 1 mM EDTA, 1% NP-40, 5% glycerol; pH 7.4) supplemented with cOmplete mini Protease Inhibitors. After homogenization, lysates were frozen in liquid nitrogen, thawed, then centrifuged twice at 5,000 ***g*** to pellet exoskeletal debris. Supernatants were collected and diluted to 1 μg/μl, and 400 μg were loaded onto 25 μl of gel pre-coupled with 10 μg of anti-GFP. A negative control was performed using uncoupled gel and *dTPI*^*+*^*/dTPI*^*Δcat*^ lysate. Samples were incubated overnight at 4°C and washed ten times with IP Lysis buffer at 4°C. Beads were eluted by boiling with 70 μl of 2X SDS–PAGE sample buffer, separated via SDS-PAGE, immunoblotted, and analyzed as outlined above. Coimmunoprecipitations were performed three times.

### FM1-43 imaging experiments

Images were taken with an Olympus BX51WI fluorescence microscope with Till Photonics Polychrome V monochromator excitation, and Hamamatsu C4742-95 digital camera. Heterozygous *TPI*^*T73R*^ larvae were maintained over TM6B, and *Tb*^*+*^ 3^rd^-instar larvae selected for analysis. Dissection and preparation of larval NMJs were performed as described previously [46]. FM1-43FX dye [Molecular Probes, Invitrogen] loading was performed as previously detailed [47]. Briefly, animals were dissected in ice cold 0 mM Ca^2+^ HL-3 with 0.5 mM EGTA, motor neurons severed, and the preps heated to room temperature or 38°C over the course of 3 min. Bath temperature was monitored throughout the experiments with a microthermal probe to ensure consistency [Fisher Scientific]. Loading experiments were performed with room temperature or 38°C preheated 90 mM KCl, 1.5 mM CaCl_2_ HL-3 supplemented with 4 μM FM1-43FX, and preparations were washed quickly and thoroughly during the experiments to avoid Ca^2+^ chelation. After loading, preparations were washed with 15 ml of 0mM Ca^2+^ HL-3 with 0.5 mM EGTA at room temperature for 10 min. Unloading experiments were performed as follows: preparations were loaded for 3 min at room temperature; washed with 15 ml of 0mM Ca^2+^ HL-3 with 0.5 mM EGTA at room temperature; imaged; washed with 38°C 0 mM Ca^2+^ HL-3; heated to 38°C over 3 min.; unloaded using 38°C 90 mM KCl, 1.5 mM CaCl_2_ HL-3 for 30 sec.; and the same synapse was imaged again.

Preparations were imaged with a water immersion 60X objective, using 450 nm excitation and a 500 nm longpass filter [Chroma Technology]. Simple PCI imaging software was used for acquisition and ImageJ for analysis. Two NMJs from muscles 6/7 were assessed per animal, one from segment A2 and A3. Six animals were assessed per genotype per time point for a total of 12 NMJs per experimental condition. After acquisition, images were relabeled by an independent researcher and blinded analysis was performed on the raw images. Boutons were outlined and intensity measured, with background subtracted from adjacent tissue. Pair-wise analyses were performed using a two-tailed Student’s t test, while comparisons among multiple experimental conditions were performed using a one-way Analysis of Variance (ANOVA) with Tukey’s post-hoc analysis.

### NMJ morphological analyses

For NMJ morphological analyses, 3^rd^-instar larvae were collected and dissected as detailed above without transection of the descending motor neurons. Preparations were fixed in 3.5% paraformaldehyde HL-3, permeabilized with 0.1% Triton X-100 in 1X PBS (PBST), and blocked with 0.2% BSA in PBST (PBSTB) for 2 hrs at room temperature. Preps were washed and incubated with goat anti-HRP [Jackson Laboratories] at 1: 200 in PBSTB for 2 hrs at room temperature. Primary antibodies were removed, washed in PBSTB, and incubated with Cy3-labeled donkey anti-goat in PBSTB at 1: 400 for 1.5 hrs at room temperature. Preps were washed, mounted in VectaShield [Vector Laboratories], and imaged within three days. Images were acquired with an Olympus confocal FV1000 microscope, using a 559 nm excitation laser. Z stacks of segment A2 of muscle 6/7 were taken using 1 μm steps. The Z stacks were merged using Olympus FV1000 Fluoview Viewer, and morphology determined. Ten animals were assessed per genotype, one NMJ per animal, for a total of ten NMJs per experimental condition. Boutons were defined as varicosities at least 2 μm in diameter, and branches defined as extensions containing at least 2 boutons. Images were relabeled by an independent researcher for blinded analysis. Variance within the data set was examined using a one-way ANOVA, with comparisons made using Tukey’s post hoc test.

### Image analysis and presentation

All image quantification was performed on raw image files acquired below saturation. Representative images were selected on the basis of raw image measurements, and post-acquisition processing was performed uniformly with grouped images in parallel using ImageJ; in agreement with published guidelines [94].

### Crystallization and structure determination of hTPI^Δcat^

Recombinant hTPI^Δcat^ containing the K13M substitution was expressed and purified as previously described [18] using affinity, anion exchange, and size exclusion chromatography. Purified protein was dialyzed into a buffer containing [20 mM Tris pH 8.8, 25 mM NaCl, 2.0% glycerol and 1 mM β–mercaptoethanol], and concentrated to 6 mg/ml prior to crystallization. Crystals of TPI^Δcat^ were obtained using the vapor diffusion method with sitting drops containing 1 μl of protein and 2 μl of well solution [28–34% PEG2000 MME, 50 mM KBr]. Initial crystals grew within 3 days and were improved by successive rounds of microseeding. Crystals were cryoprotected in 40% PEG 2000MME, 20% glycerol, 50 mM KBr, prior to flash freezing in liquid nitrogen.

Data collection was performed at the National Synchrotron Light Source at beamline X25 and using a Pilutas 6M detector. Diffraction data was integrated, scaled, and merged using HKL2000 [95]. hTPI^Δcat^ crystals belong to space group P2_1_2_1_2_1_ and contain a dimer in the asymmetric unit. Initial phases were estimated for hTPI^Δcat^ via molecular replacement using a previously determined structure of wild-type as our search model [18]. Model bias was reduced through simulated annealing and the model was further improved by manual model building combined with positional and anisotropic B-factor refinement within Phenix [96]. Model quality was validated using MolProbity [97]. Figs were generated using PyMOL (PyMOL Molecular Graphics System, Schrödinger, LLC). Coordinates and structure factors for hTPI^Δcat^ have been deposited within the Protein Databank under accession code 4ZVJ.

### Molecular modeling of dimer interface substitutions

The effect of hT75R and hG76E substitutions were modeled onto hTPI^Δcat^ or hTPI^WT^ structural templates using the RosettaBackrub analysis as implemented within the RosettaBackrub server [98]. For all predictions, Rosetta version 3.1 was used as the algorithm with a backrub radius of 15 Å to ensure that perturbations extending away from the site of the substitution could be sampled. An ensemble of 50 structures was predicted for each TPI model, and their reported Rosetta scores normalized to the starting template. Structures whose Rosetta scores were in the most favorable 10% of the ensemble were used in Fig 9D.

### TPI nomenclature

Triosephosphate isomerase research has been split between those studying the enzymatic and structural properties of the protein, and those studying its role in disease. Researchers focusing on the pathology of TPI deficiency typically use the abbreviation “TPI”, whereas enzymologists and structural biologists used the abbreviation “TIM”. The aim of this study was to determine the molecular mechanism of a disease mutation, and as such we have used the abbreviation “TPI”.

## Supporting Information

S1 Fig *hTPI*^*M82T/null*^ phenocopies the behavioral and longevity effects of *dTPI*^*M80T/null*^. (A) *dTPI*^*M80T/null*^ and *hTPI*^*M82T/null*^ elicit significant delays in time to recovery from mechanical stress at Day 3, (B) time to paralysis after thermal stress at Day 4, and (C) longevity compared to *dTPI*^*WT/null*^ and *hTPI*^*WT/null*^, respectively. All animals were reared at 25°C, and thermal stress paralysis times at 600 sec. represent wild type behavior; the assay was stopped at 10 min. as previously outlined for *hTPI* alleles [18]. n≥20 for behavior and n≥70 for lifespans. The *TPI*^*null*^ and *hTPI*^*WT*^ alleles were generated previously [18,19], with *TPI*^*null*^ representing a deletion of two of three constitutive *TPI* exons. Comparisons were made with Student’s T test, and lifespans by a Log-rank (Mantel-Cox) survival test, * indicates p<0.05 and *** p<0.001.
(EPS)

S2 Fig TPI dimer interface substitutions impair enzyme dimerization. Purified hTPI^Δcat^, hTPI^T75R^, hTPI^G76E^, and hTPI^T75R,G76E^ were examined via size-exclusion chromatography for monomer and dimer content. Each dimer interface substitution displays a significant increase in monomeric TPI relative to the enzyme dimer (n≥3). hTPI^WT^ and hTPI^Δcat^ intermittently displayed some monomer species, and were prepared and analyzed in parallel with the dimer mutations. Comparisons were made with a One-way ANOVA using Tukey’s post hoc test, * indicates p<0.001.
(EPS)

S3 Fig RNAi knockdown of *TPI*^*WT*^ fails to recapitulate *dTPI*^*M80T*^ phenotypes. (A) TPI protein levels were knocked down ubiquitously and confirmed in thorax and (B) head tissues (n≥3) with quantification and representative images. (C) Knockdown animals failed to display typical mechanical-stress dependent paralysis at Day 5 aged at 25°C (n≥15). *TPI*^*WT*^ animals are *w*^*1118*^*;;* controls while the *TPI*^*M80T*^ allele used was formerly known as *TPI*^*sgk*^ [19]. Comparisons were made with a One-way ANOVA using Tukey’s post hoc test, *ns* indicates no significance, ** p<0.01, and *** p<0.001 relative to WT. Experiments shown are derived from knockdown using VDRC line #25644; VDRC #25643 was also examined, with similar results.
(EPS)

S4 Fig *TPI*^*T73R*,*G74E*^ behavioral dysfunction and longevity. (A) *dTPI*^*T73R*,*G74E*^ is characterized by severe mechanical and (B**)** thermal stress sensitivity, which respond positively to complementation by *dTPI*^*Δcat*^. Thermal stress paralysis times at 360 sec. represent wild type behavior; the assay was stopped at 6 min. (C) *TPI*^*T73R*,*G74E*^ exhibits reduced lysate catalytic activity compared to WT. (D) *TPI*^*Δcat*^ fails to complement *TPI*^*T73R*,*G74E*^ longevity. n≥30 for all behavior, n≥80 for all lifespans. Comparisons were made with a One-way ANOVA using Tukey’s post hoc test, and lifespans by a Log-rank (Mantel-Cox) survival test, *ns* indicates no significance, ** p<0.01, *** p<0.001.
(EPS)

S5 Fig Lysate isomerase activity does not predict disease presence or severity. (A) Lysate activity is indicated and (B) expanded as per the dashed box. Comparisons were made with a One-way ANOVA using Tukey’s post hoc test, *ns* indicates no significant differences and *** p<0.001. Biological replicates are indicated.
(EPS)

S6 Fig C-terminal CFP tags do not alter TPI dimer mutant neuropathology. C-terminal CFP tags were added to dTPI^T73R^, dTPI^G74E^, and dTPI^T73R,G74E^. (A) Animals were crossed with similar alleles lacking the CFP tag, and the resulting progeny retain the mechanical- and (B) thermal-stress sensitivity. n>10. Thermal stress paralysis times at 360 sec. represent wild type behavior; the assay was stopped at 6 min. Comparisons were made with a One-way ANOVA using Tukey’s post hoc test, *** indicates p<0.001. # indicates the animals did not paralyze within 360 sec.
(EPS)
